# Bridging consent: from toll bridges to lift bridges?

**DOI:** 10.1186/1755-8794-4-69

**Published:** 2011-10-04

**Authors:** Isabelle Budin-Ljøsne, Anne Marie Tassé, Bartha Maria Knoppers, Jennifer R Harris

**Affiliations:** 1 Norwegian Institute of Public Health, Division of Epidemiology, Department of Genes and Environment, P.O Box 4404 Nydalen, NO-0403 Oslo, Norway; 2 Centre of Genomics and Policy, McGill University, 740 Dr Penfield Avenue, suite 5202, Montreal (Quebec) H3A 1A4, Canada

## Abstract

**Background:**

The ability to share human biological samples, associated data and results across disease-specific and population-based human research biobanks is becoming increasingly important for research into disease development and translation. Although informed consent often does not anticipate such cross-domain sharing, it is important to examine its plausibility. The purpose of this study was to explore the feasibility of bridging consent between disease-specific and population-based research. Comparative analyses of 1) current ethical and legal frameworks governing consent and 2) informed consent models found in disease-specific and population-based research were conducted.

**Discussion:**

Ethical and legal frameworks governing consent dissuade cross-domain data sharing. Paradoxically, analysis of consent models for disease-specific and population-based research reveals such a high degree of similarity that bridging consent could be possible if additional information regarding bridging was incorporated into consent forms. We submit that bridging of consent could be supported if current trends endorsing a new interpretation of consent are adopted. To illustrate this we sketch potential bridging consent scenarios.

**Summary:**

A bridging consent, respectful of the spirit of initial consent, is feasible and would require only small changes to the content of consents currently being used. Under a bridging consent approach, the initial data and samples collection can serve an identified research project as well as contribute to the creation of a resource for a range of other projects.

## Background

Millions of human biological samples and associated health data are stored in research biobanks and constitute essential resources for biomedical researchers interested in accessing large and valuable datasets needed to study complex disease aetiology, improve diagnostics, and advance the agenda of personalized medicine. The ability to share and combine data from disease-specific and population-based research has become increasingly important for translational medicine. For instance, research designs that recruit by genotype rather than by illness outcome [1] are becoming more common and will also be a fruitful approach for studying pathways to disease where variation along the continuum from pre-disease status to clinical manifestation is analyzed. Such study designs will entail extensive cross-domain data sharing between disease-specific and population-based research. However, cross-domain data sharing between these different research environments remains limited, partly because such sharing is not anticipated in most traditional consent forms. This is particularly true in disease-specific clinical research settings where informed consent traditionally is targeted to research purposes related to a specific disease and does not anticipate or accommodate future research aims that could be addressed with the data [2,3]. In this paper, we investigate whether it is possible and feasible to bridge consent, that is to formulate consent models that include the necessary core elements for each research domain and simultaneously anticipate cross-domain data sharing.

To investigate this, we conduct two sets of comparative analyses that focus on: 1) current ethical and legal frameworks governing consent with regards to potential hindrances and opportunities for cross-domain sharing, and 2) current consent models found in disease-specific and population-based research. The purpose of this work is to identify potential hindrances and opportunities to bridge consent and to propose practical solutions to bridging consent that dovetail with the new interpretation of consent as envisioned by the Human Genome Organization's Ethics Committee.

For the purpose of this study, we identified three separate domains of research across which data sharing would be valuable: 1) Disease Specific Biobank Research (DSBR) refers to research typically conducted by large research consortia outside of a clinical setting using a collection of human biological material and associated information stored for research on a pre-determined set of diseases; 2) Disease Specific Clinical Research (DSCR) refers to clinically-based research conducted in association with a biobank to evaluate the effectiveness and safety of medications or medical devices (e.g. clinical trials); 3) Population Biobank Research (PBR) refers to research conducted on populations unselected with respect to a particular disease using human biological material and associated information stored for future unspecified research.

First, a comparative analysis of relevant ethical and legal frameworks governing consent in biobanking was conducted with regards to identifying potential hindrances and opportunities for cross-domain sharing. Regulatory consent requirements regarding the content of consent forms were not analysed as each country has different ethical and legal norms. Relevant international documents and guidelines were identified with the HumGen International database [4] and official websites of the Council of Europe [5]. Variations of the following keywords were used to guide our searches, either alone or in conjunction: ['research'] and/or ['samples' and/or 'data' and/or 'information'] and/or ['consent'] and/or ['genetics' and/or 'medical' and/or 'health'] and/or ['biobank' and/or 'hospital' and/or 'repository' and/or 'file']. This review is based upon documents written in or translated into English or French, before March 1st, 2011.

Next, to examine the feasibility of bridging consent, a comparative analysis of consents in the three research environments was conducted. To do so, three sources of information were examined: 1) consent forms referred to as consent models and made publicly available by international organisations and research consortia, 2) consent models proposed in the literature, and 3) elements of consent recommended in guidelines from international organisations and councils. An internet search was conducted to identify the information in point 1 and 3 above by using the following key words: ['informed'] and ['consent'] and ['model' and/or 'template']. A similar search was made in Pubmed to identify models of consent form proposed in the literature. The criteria used to select the consent requirements were that the consent models or recommended elements of consent were developed by organisations and research consortia that are representative of their research domain due to their size, international profile and recognition or that they were developed through research analyzing informed consent materials used in a specific research domain [6-22]. Our comparative analysis identified 1) core elements in the *structure *of the consents, and 2) similarities and differences in the information *content *of consents.

## Discussion

### Results from the comparative analysis of ethical and legal frameworks governing consent

The results, based upon a comparative analysis of whether ethical and legal frameworks governing consent provide opportunities for bridging, show that the bridging of consent between different research domains is not specifically addressed. Consent to research is based on an extensive set of international and national ethics guidelines which have always required the respect for the autonomy of research participants via a free and informed consent. Classical informed consent requires the appropriate disclosure of the specific research objectives, procedures, risks, and benefits to participants [7,8,22-28]. The use of biological samples and data outside the range of the research of the consent form would in these guidelines be considered a 'secondary use', which is neither legal nor ethical in most jurisdictions, in the absence of a new consent, an ethics waiver, or legal provisions [29]. Therefore, the use of already collected samples and data in different research domains appears to be nearly impossible.

Our analysis identified one exception to this restrictive view of consent. In 1995, the Human Genome Organization's Ethics Committee stated, in the *Statement on Human Genomic Databases *[30], that the choices of individuals should be respected, informed consent may include notification of uses (actual or future) or opting out, and mechanisms should be established to ensure respect for such choices. This statement indicates the possibility to share a participant's samples, data, and results from one research setting to another, as long as the choices expressed by the participant in the initial research are respected. This guideline is the first step into a paradigm shift towards a more flexible interpretation of consent. Such paradigm shift is necessary to allow the bridging of consents which, on the surface, would not allow uses of samples or data in other research domains. Therefore, in order to respect the choices expressed in the initial consent, it could be assumed that a research participant would agree to allow his/her samples, associated data, and results to be accessed for other research in different research domains, provided that the new research focuses on the same diseases or a more restrictive set of diseases than the initial research.

### Results from the comparative analysis of consent models

The results of our comparative analysis of consent models across the three different research domains (DSBR, DSCR and PBR) reveals considerable congruence regarding consent requirements. The models for all three domains share the same consent structure comprised of 15 core elements. This similarity most likely arises because informed consent models used in DSBR and PBR are derived from those used in DSCR [14] and because protection of the participant rights is the fundamental concern of all consents. Our analysis also shows that the nature of the information provided to research participants under each core element is highly similar (See Table 1 - Differences and Similarities in Informed Consent Requirements in DSBR, DSCR and PBR). However, differences exist in terms of length, scope and level of detail of the information provided in the consents as described in more detail below. Elements of consent in which no differences could be identified are not listed.

**Table 1:** Differences and Similarities in Informed Consent Requirements in DSBR, DSCR and PBR

Element 1	Invitation, presentation of the study and voluntary participation
**Similarities**	Study title and location Names and affiliations of principal investigators and co-investigators Study type, purposes and justification Criteria for participant selection and estimated number of participants Sources and duration of funding Voluntary participation

**Differences**	**Found in DSCR, not in DSBR and PBR** Description of clinical trial Reasons for testing Current experience with drug/device Availability of products and interventions resulting from the research

**Element 2**	**Research protocols**

**Similarities**	Duration and timetable of the study Procedures (physical exams, clinical measurements, samples intake, number of visits, interventions) Types of data and samples to be collected

**Differences**	**Found in DSCR, not in DSBR and PBR** Description of routine, experimental clinical procedures (randomization, blinding, placebo use, biopsy, surgery, etc.) Expected duration of participation in the study Circumstances of early termination **Found in DSBR and PBR, not in DSCR** Re-contact procedures

**Element 3**	**Risks**

**Similarities**	Known and anticipated physical, emotional and psychological risks and discomforts Known and anticipated risks associated with breaches of confidentiality Medical procedures in the event of harm or in the need of treatment of research-related injuries

**Differences**	**Found in PBR, not in DSCR and DSBR** Risks of potential group stigmatization

**Element 4**	**Benefits**

**Similarities**	Expected direct and indirect benefits to participants, local community and broader scientific community Information about return of personal or financial benefits to participants (if any)

**Differences**	**Found in PBR, not in DSCR and DSBR** Scientific importance of biobanks for the public good

**Element 5**	**Financial compensation, indemnification and costs**

**Similarities**	Procedures for incentives and financial compensations

**Differences**	**Found in DSCR, not in DSBR and PBR** Costs in case of treatment of research-related injuries Costs related to participation in research

**Element 6**	**Confidentiality**

**Similarities**	Procedures for data protection and maintenance Limits to confidentiality protection

**Differences**	**Found in DSBR and PBR, not in DSCR** Prohibition of data re-identification

**Element 7**	**Data access and data sharing**

**Similarities**	Conditions and procedures for internal and external access to data, biological samples and health records (e.g. by researchers, participants or regulatory bodies) Conditions for secondary or future uses of data and biological samples within the same research area

**Differences**	**Found in DSCR, not in DSBR and PBR** Participant's right to place restrictions on specific future uses of data and biological samples **Found in PBR, not in DSCR and DSBR** Requirements for return of samples and research results to the biobank

**Element 8**	**Storage**

**Similarities**	Procedures for storage and destruction of data and samples, also in the case of participant withdrawal

**Differences**	**Found in PBR, not in DSCR and DSBR** Procedures for use of data and samples after donor's death Rules regulating the use of data and samples in case of biobank or owner/custodian surrender

**Element 9**	**Return of results**

**Similarities**	Procedures for individual return of results to participants/medical records during or after the research

**Differences**	**Found in DSBR and PBR, not in DSCR** Procedures for return of general results by means of a Newsletter, web site etc.

**Element 10**	**Commercialization**

**Similarities**	Prohibition of samples commercialization Potential for third-part commercialization of products resulting from the research Non return of financial benefits to the participants

**Differences**	**Found in DSBR, not in DSCR and PBR** Rules for ownership of samples

**Element 11**	**Right to withdraw**

**Similarities**	Participant's right to withdraw at any time without penalty or loss of benefits

**Differences**	**Found in DSCR, not in DSBR and PBR** Alternatives to participation Description of established standard treatments **Found in DSBR and PBR, not in DSCR** Placement of data in open or controlled databases Impossibility to withdraw data already contained in analyses

**Element 12**	**Re-contact**

**Similarities**	Re-contact procedures (e.g. frequency, method) if any

**Differences**	**Found in DSBR and PBR, not in DSCR** Re-contact procedures for new unanticipated research uses

**Element 13**	**Contact information**

**Similarities**	Contact information for principal investigators, co-investigators and sponsoring institutions Contact information for the Institutional Review Board which granted approval

**Element 14**	**Ethics oversight**

**Similarities**	Information about procedures for ethics oversight of current project

**Differences**	**Found in DSBR and PBR, not in DSCR** Information about procedures for ethics oversight of future projects

**Element 15**	**Signatures**

**Similarities**	Signatures of participant, witness (if the participant is illiterate), principal investigators and translator (if needed)

### Invitation, presentation of the study and voluntary participation

In the research settings studied the consent forms begin with an invitation to participants and a brief presentation of the research (study's title, name and affiliation of the principal investigator and co-investigators). Information about the voluntary nature of participation in the research is usually provided. However, informed consent models for DSCR emphasize the specific research goals of the study in their introductory information (i.e. trial of a new treatment for breast cancer) while models in DSBR and PBR, where the research goals are broader (i.e. cancer research or research on common diseases affecting populations) focus more on explaining that the data and samples collected will be used for research related to a specified disease or group of diseases.

### Research protocols

Consent forms in the three research settings describe the research protocol, including the type of samples and data to be collected, the nature of the interventions (if any), the duration and timetable of the study, and circumstances under which early termination may occur. If the original research setting is clinical research, this section also provides information about the clinical procedures and randomization. Procedures are widely described in DSCR while they are less described in DSBR and PBR where it is more typical to find general descriptions regarding the type of data and samples that will be collected and regarding re-contact procedures that may be necessary during the conduct of the study.

### Risks

In all consent forms the section on risks describes known and anticipated physical, psychological and informational risks related to participation in the study. Procedures that will be undertaken if risks materialize are also described. Models in DSCR place emphasis on the physical and psychological risks for the participant and their relatives while models in DSBR and PBR place emphasis on informational risks (e.g. risks associated with breaches of confidentiality). Potential group harm in the event of breaches of confidentiality is only mentioned in PBR.

### Benefits

Consents under all three research settings include information about the expected direct and indirect benefits for the participants, the local community and the broader scientific community. DSCR emphasizes direct benefits to the research participants while DSBR and PBR emphasize indirect benefits to larger groups and highlight the scientific relevance of biobanks.

### Financial compensation, indemnification and costs

Consent forms in all three research settings provide information about costs related to participation in the study and financial compensation (if any) offered to the participants. In DSCR, information about treatment costs and research-related injury costs is provided, which is not the case in DSBR and PBR.

### Confidentiality

Consent forms in all three research settings describe procedures ensuring the protection and security of data and samples, the privacy of research participants and the confidentiality of their data. These procedures are briefly mentioned in DSCR while they are described in more depth in DSBR and PBR where e.g. the risk for re-identification is mentioned as a potential issue.

### Data access and data sharing

All three research domains provide information regarding conditions and procedures for internal and external sharing of data, samples and results. DSCR emphasizes informing research participants about data sharing restrictions. Data sharing for future research with other researchers is usually not mentioned. In contrast, data sharing procedures are widely described in DSBR and PBR. It should be noted that it is only in PBR that information is given about the requirement to return samples and research results to the biobank from which they originate.

### Storage

All three research domains provide information about data and sample retention, storage and destruction (also in the case of participant withdrawal) although it is more comprehensive in DSBR and particularly in PBR where information about the fate of the data and samples in the event of donor's death or biobank dismantling is provided.

### Return of results

All three research settings provide information about procedures for feedback of general and/or individual results during and after the study (if any) and the inclusion of study results in the medical record. Overall strategies for communication with participants and society are also described. While procedures for individual feedback of results are usually described in details in DSCR, they are described in shorter terms in DSBR and PBR where individual return of results are either not practised or practised restrictively (e.g. only clinical measurements are returned).

### Commercialization

All three research domains provide information about samples not being used for commercial purposes, potential third-part commercialization and policies of no return of financial benefits to research participants. The issue of sample ownership is addressed in DSBR while it is not mentioned in DSCR and PBR.

### Right to withdraw

Information about the right to withdraw remains uniform through all three research domains although more detailed information about e.g. the fate of data and samples in the case of participant withdrawal is provided in PBR.

### Re-contact

Consent forms in all three research settings inform participants about re-contact procedures when investigators for instance need to collect additional data or obtain authorization to conduct new research on the data and samples collected. Re-contact procedures for new unanticipated research uses are only mentioned in DSBR and PBR.

### Ethics oversight

Information about procedures for ethics oversight is provided in all three types of research although ethics oversight of future projects is only mentioned in DSBR and PBR.

## Summary

We analyzed the feasibility of bridging consent between disease-specific and population-based research. The results of our comparative analysis of ethical and legal frameworks governing consent show that the current interpretation of consent in existing international and national ethics guidelines does not facilitate data sharing in general. Paradoxically, the results of our comparative analysis of actual consent requirements reveal no significant differences across the research domains studied. Following in the footsteps of the Human Genome Organization's Ethics Committee, the adoption of a new interpretation of consent, based on the spirit of the initial consent given by the participant, would facilitate cross-domain data sharing though the bridging of consent across research domains. Such bridging would be possible as long as the choices expressed by the participant in the initial research are respected. It could for instance be assumed that a research participant would agree to allow his/her samples, associated data, and results to be accessed for other research, in different research domains, provided that the new research focuses on the same diseases or a subset thereof, stipulated in the initial research. In the future, attention may shift away from disease endpoints as more focus is placed on pathways to disease [31]. However, as long as international guidelines require research subjects to be informed of the disease or set of diseases under study, it seems ethically hazardous to overly stretch an already restricted consent. Therefore, where initial consent is restricted to a specific disease, we propose that bridging is possible only with other similar disease specific research (which would then be considered as a primary use of data and samples, and not a secondary use).

Our analysis of the literature and consent forms demonstrates that most consent forms could be used as the basis for a bridging consent. To allow bridging, additional information should be included under each core element of consent as described in Table 2 (Proposed Modifications that Enable Bridging Consent) to support data sharing and unspecified secondary uses of the data, the samples and the research results. Bridging consent is not expected to significantly increase the length of most of the restrictive consent forms as it requires the inclusion of only a few additional elements of information such as for example concerning sample or data sharing. It should be noted that bridging consent does not alter the requirement to obtain REB review for new research involving human subjects, data or tissues.

**Table 2:** Proposed Modifications that Enable Bridging Consent

	Disease Specific Biobank Research and Disease Specific Clinical Research
**Element 1**	**Invitation, presentation of the study and voluntary participation**

	Scientific relevance of biobanks

	Importance for research

	**Data and samples **use for other related conditions or in other research settings (such as **disease specific biobanks**)

**Element 2**	**Research protocols**

	No additional elements required

**Element 3**	**Risks**

	No additional risks, except related to expansion of scope of research

**Element 4**	**Benefits**

	Expected benefits related to the use of **data and samples **for other related conditions or other research settings (such as **disease specific biobanks**)

**Element 5**	**Financial compensation, indemnification and costs**

	No additional elements required

**Element 6**	**Confidentiality**

	No additional confidentiality issues, except related to expansion of scope of research (data storage/retention)

**Element 7**	**Data Access and data sharing**

	Access procedures to data, samples and results by other research settings

	Return of new data, samples and results from the other research settings to the original research setting

	The participant's right to agree/disagree to access to their **data and samples **for other related conditions or other research settings (such as **disease specific biobanks**)

**Element 8**	**Storage**

	Rules related to the use, retention, storage and destruction of data and samples for other related conditions or other research settings (such as **disease specific biobanks**)

**Element 9**	**Return of results**

	Return of results for research performed in other related conditions or other research settings (such as **disease specific biobanks**); (return to the original research setting, general vs. individual, return to the participant (or not), etc.)

**Element 10**	**Commercialization**

	Potential commercialization and possible patenting of study related tests, drugs, devices, etc. in other research or settings

**Element 11**	**Right to withdraw**

	Creation of a uniform process for withdrawal

**Element 12**	**Re-contact**

	Re-contact for use of samples, data and results in other research settings (such as disease specific research projects or other disease specific biobanks or population biobanks)

	Creation of a uniform process to ensure that the re-contact procedure is respected in every research setting

**Element 13**	**Contact information**

	Contact information for principal investigator, co-investigators, sponsoring institutions (when applicable) and the Institutional Review Board in other research settings

**Element 14**	**Ethics oversight**

	Information about the extent of ethics oversight and approval mechanisms for the use of the data, samples and results of this study in other research settings

**Element 15**	**Additional choices and signatures**

**Disease Specific Biobank Research**	**Add:** I agree that the samples and data collected during [*name of the study*], be used in other research settings, for research on [*name of the disease*] and related conditions or included in [*name of disease specific biobank*]. YES/NO

**Disease Specific Clinical Research**	**Add:** I agree that the samples and data collected during [*name of the study*], be used for research on [*name of the disease*] and related conditions or included in [*name of disease specific biobank*]. YES/NO

No additional elements are required for Population Biobank Research.

Bridging consent requires that the responsibilities between the original data collectors and future users of the data be clearly outlined, regarding sharing, maintenance and protection of data. Procedures for the protection of privacy, protection of the participant's right to withdraw and return of results should also be shared between the original data collectors and future users, and clear guidelines should be established. Many of these challenges are already being addressed through ELSI harmonization initiatives aiming to implement data sharing codes of conduct and policies [32] and are encouraged by empirical studies indicating that research participants generally support wide data sharing for research purposes given that privacy and confidentially concerns are appropriately handled and secured [33-35].

**Figure 1:**
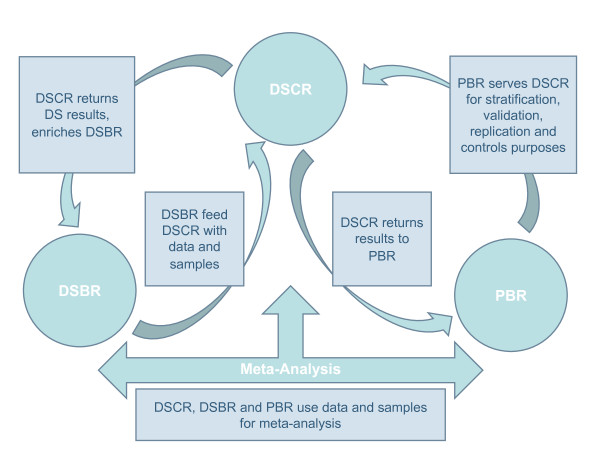
**Bridging consent: The lift bridge**.

Bridging consent has many advantages. First, it allows consent to evolve in pace with biomedical science. For many studies in bioscience today the traditional distinction between clinical and non-clinical data is becoming less relevant and it is important that the ethics-based structures put in place to support contemporary research keep apace with the changing nature of the science. Second, bridging consent has the potential to maximize the use of human biological resources according to the requirements of many funders as illustrated in Figure 1. Third, bridging consent allows economies of scale when bridged consents are used by research teams across their research programs. Finally, bridging consent may promote greater transparency around data sharing practices which are often made possible through e.g. Research Ethics Committees approval without the research participants' knowledge [3,36]. While largely applicable to the use of previously collected samples and data, the bridging of consents is the first part of a new scheme proposing a more flexible approach to the use of data collections in different research domains. However, a complete integration of research domains will probably require the creation of an international code of conduct for data sharing across research domains.

## Abbreviations

DSBR: Disease Specific Biobank Research; DSCR: Disease Specific Clinical Research; PBR: Population Biobank Research; REB: Research Ethics Board.

## Competing interests

The authors declare that they have no competing interests.

## Authors' contributions

IBL conceived and drafted the study jointly with the other co-authors. She carried out the comparative analysis of informed consent models, the interpretation of data and drafted the final version of the manuscript. AMT carried out the policy and guidelines review, the analysis and interpretation of data, as well as drafted parts of the manuscript. BMK conceived the study jointly with the other co-authors and participated in its design and coordination. BMK helped to draft the manuscript and added comments and suggestions to the manuscript.

JRH conceived the study jointly with the other co-authors and participated in its design and coordination. JRH helped to draft the manuscript and added comments and suggestions to the manuscript. All authors read and approved the final manuscript.

## Authors' information

Isabelle Budin-Ljøsne, BA, is an ethics adviser and training coordinator for the European collaborative research project ENGAGE. She is involved in the Biobank Norway project funded by the Norwegian Research Council and the BioSHaRE-EU project funded by the European Commission. Isabelle's focus is on addressing the ethical, legal and social questions relevant to molecular genetics, genomics and its translation to the clinic.

Anne Marie Tassé, LL.B., LL.M., M.A., LL.D(c), is a lawyer specialised in health law and bioethics. Specialised in international comparative law, her work looks primarily at interactions between law and ethics, in the areas of biomedical research and genetics. She also works with national and international research consortia.

Bartha Maria Knoppers, PhD, is Director of the Centre of Genomics and Policy, Faculty of Medicine, Department of Human Genetics, McGill University. Canada Research Chair in Law and Medicine, she held the Chaire d'excellence Pierre Fermat (France) (2006-2008) and was named Distinguished Visiting Scientist (Netherlands Genomics Initiative) (2009-2011).

Jennifer R. Harris, PhD, is a senior researcher in the Department of Genes of Environment, Division of Epidemiology at the Norwegian Institute of Public Health (NIPH) in Oslo. Her training is in life-span development and genetics. She is active in several EU-biobanking projects including the new project BioSHaRE-EU that starts in 2011.

## Pre-publication history

The pre-publication history for this paper can be accessed here:

http://www.biomedcentral.com/1755-8794/4/69/prepub
